# Current status of transcatheter mitral valve replacement: systematic review and meta-analysis

**DOI:** 10.3389/fcvm.2023.1130212

**Published:** 2023-05-10

**Authors:** Alberto Alperi, Pablo Avanzas, Victor Leon, Iria Silva, Daniel Hernández-Vaquero, Marcel Almendárez, Rut Álvarez, Félix Fernández, Cesar Moris, Isaac Pascual

**Affiliations:** ^1^ Department of Cardiology, Heart Area, Hospital Universitario Central de Asturias, Oviedo, Spain; ^2^ Health Research Institute of Asturias (Instituto de Investigación Sanitaria del Principado de Asturias), Oviedo, Spain; ^3^ Department of Medicine, Faculty of Medicine, University of Oviedo, Oviedo, Spain

**Keywords:** mitral valve, mitral regurgitation, mitral insufficiency, mitral incompetence, transcatheter mitral valve replacement

## Abstract

**Introduction:**

Mitral Regurgitation (MR) has a strong impact on quality of life and on mid-term survival. Transcatheter mitral valve replacement (TMVR) is rapidly expanding and a growing number of studies have been published recently.

**Methods:**

A systematic review of studies reporting on clinical data for patients with symptomatic severe MR undergoing TMVR was performed. Early- and mid-term outcomes (clinical and echocardiographic) were evaluated. Overall weighted means and rates were calculated. Risk ratios or mean differences were calculated for pre- and post-procedural comparisons.

**Results:**

A total of 12 studies and 347 patients who underwent TMVR with devices clinically available or under clinical evaluation were included. Thirty-day mortality, stroke and major bleeding rates were 8.4%, 2.6%, and 15.6%, respectively. Pooled random-effects demonstrated a significant reduction of ≥ grade 3+ MR (RR: 0.05; 95% CI: 0.02–0.11; *p* < 0.001) and in the rates of patients in NYHA class 3–4 after the intervention (RR: 0.27; 95% CI: 0.22–0.34; *p* < 0.001). Additionally, the pooled fixed-effect mean difference for quality of life based on the KCCQ score yielded an improvement in 12.9 points (95% CI:7.4–18.4, *p* < 0.001), and exercise capacity improved by a pooled fixed-effect mean difference of 56.8 meters in the 6-minute walk test (95% CI 32.2–81.3, *p* < 0.001).

**Conclusions:**

Among 12 studies and 347 patients comprising the updated evidence with current TMVR systems there was a statistically significant reduction in ≥ grade 3+ MR and in the number of patients exhibiting poor functional class (NYHA 3 or 4) after the intervention. Overall rate of major bleeding was the main shortcoming of this technique.

## Introduction

Mitral regurgitation (MR) is a prevalent valvular heart disease in western countries (1) and, when left untreated, leads to dilation and dysfunction of the left ventricle, as well as to heart failure decompensation and a poorer long-term prognosis (2). Open heart surgery has been the treatment of choice in MR patients for decades. However, many patients are deemed unsuitable for cardiac surgery based on comorbidities and surgical risk (3). Hence, less invasive percutaneous techniques have appeared in order to overcome this unmet clinical need. Catheter-based therapies mimicking surgical mitral valve repair face some limitations like their applicability on difficult anatomies (e.g., calcified mitral apparatus, short and restrictive posterior mitral leaflet) and the recurrence of significant MR overtime (4). Transcatheter mitral valve replacement (TMVR) constitutes a less invasive approach compared to open heart surgery, and it may help overcome some of the limitations associated with transcatheter edge-to-edge repair (TEER). Several dedicated TMVR devices for the mitral position are under clinical evaluation (5), and a growing body of evidence for this therapy is currently available.

In this systematic review and meta-analysis, we aimed to delineate pooled clinical outcomes observed for this technique using current TMVR systems, and to provide data on MR reduction and patientś clinical improvement after the intervention.

## Methods

A comprehensive systematic review of published studies reporting on patients with significant MR who received TMVR was performed following the specified on the Preferred Reported Items for Systematic Reviews and Meta-Analysis (PRISMA) statement (6).

A computer-based search was performed on PubMed and EMBASE databases in order to identify relevant studies, and manual search of references from the selected studies (backward snowballing) was also used. Reviews, meta-analyses, and editorials were also reviewed in order to identify other potential entries.

The following keywords were used: “transcatheter mitral valve replacement” and “transcatheter mitral valve implantation.” The last access to databases was on October 31st 2022.

Eligible studies were those of original design, which were published in English, and that reported on clinical outcomes after TMVR with the use of dedicated devices for the mitral position. When the same patient population was used for several reports, the study with the largest sample size was finally selected. Studies including patients who underwent simultaneous TMVR and other valve interventions were excluded, as well as single case reports and congress communications. Studies informing on the use of transcatheter valves intended for the aortic position in patients with severe mitral annular calcification, a prior mitral annuloplasty, or a failed surgical bioprosthetic valve were also excluded.

A standardized data abstraction sheet was used for data extraction. Clinical characteristics, in-hospital, 30-days and mid-term outcomes were collected as reported by authors. Two investigators (A.A and I.P.) performed the literature search, study selection, and data acquisition. Any discrepancies were solved by a third investigator (P.A.).

### Endpoints

The outcomes evaluated in this study were: (i) 30-day complications (all-cause mortality, stroke, life-threatening/major bleeding, acute kidney injury and conversion to open heart surgery), (ii) 30-day rate of ≥ grade 3+ MR, changes in New York Heart Association (NYHA) functional class and in quality of life and exercise capacity evaluated by the Kansas City Cardiomyopathy Questionnaire (KCCQ) and 6-minute walk test, respectively; and (iii) mid-term outcomes (mortality, heart failure hospitalization, and NYHA functional class).

### Statistical analysis

Continuous variables are displayed as mean (standard deviation) and categorical variables as frequencies (percentages) when presenting baseline characteristics. Risk ratio (RR) or mean difference (MD) and their 95% confidence intervals (CI) were calculated for the following endpoints aiming to compare the results before and after procedure: ≥grade 3+ MR, NYHA class 3 or 4 at 30-day follow-up, and KCCQ quality of life score. The I^2^ index was utilized to assess consistency between studies. This index takes values between 0% and 100%, and the thresholds frequently used for heterogeneity are as follows: 25% suggests low, 50% moderate, and 75% large heterogeneity (7). Fixed-effects model was utilized for calculating pooled estimates in case of low heterogeneity, whereas a fixed-effects model was used in case of significant heterogeneity. The Egger regression and visual inspection of funnel plots were used for the assessment of publication bias.

Weighted means (95% confidence interval) or frequencies (percentages) were used for overall outcomes and pooled baseline characteristics. For calculating means and standard deviation when medians and interquartile ranges were provided we used the formula from Wan et al. (8). Weighted means were calculated based on the total number of patients in each study (weight = *n*).

The analyses were performed using the following software: STATA (v14.0; StataCorp) and Review Manager version 5.4 (The Nordic Cochrane Centre, The Cochrane Collaboration).

## Results

### Study selection

**Figure 1:**
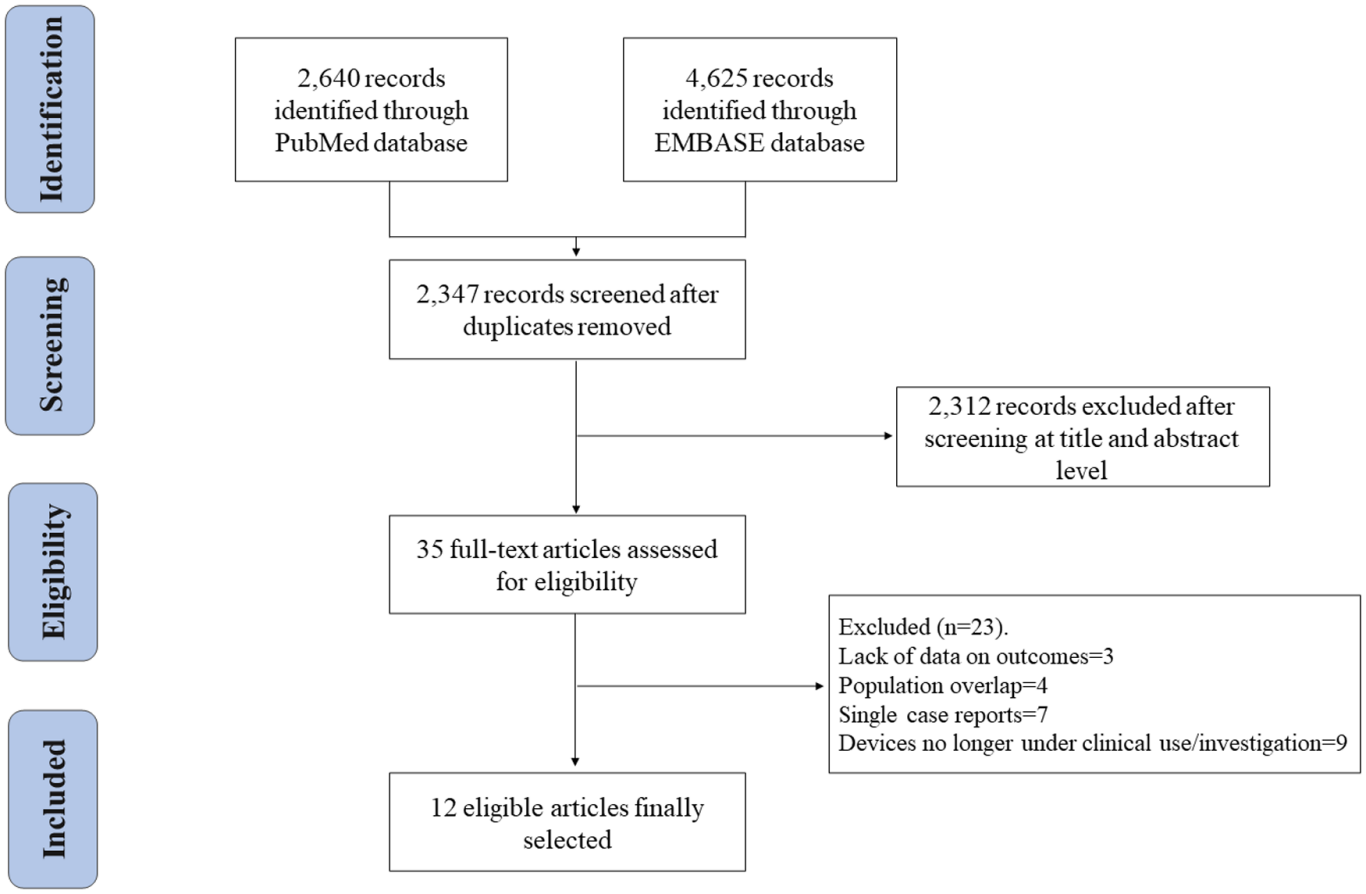
Flow chart, based on the preferred reported items for systematic reviews and meta-analysis (PRISMA) statement, of studies selected assessing early- and/or mid-term outcomes after transcatheter mitral valve replacement.

A total of 2,640 and 4,625 records were identified in the searches from PUBMED and EMBASE, respectively. This yielded 2,347 records which, after exclusion of duplicates, were reviewed at title and abstract level. From them, 31 articles were selected and their full text was assessed for eligibility. Finally, a total of 12 studies fulfilled the inclusion criteria and were selected for the final analysis (9–20): 7 observational studies and 5 single-arm clinical trials. The PRISMA flow-diagram is displayed in Figure 1, and the main features of the studies included are shown in Table 1.

**Table 1:** Characteristics of the included studies for TMVR.

Study	Number of patients	Number of centers	Study design	Device(s)	Main access	Follow-up	Exclusion criteria
Alperi et al. 2020 (9)	3	1	Observational (compassionate use)	Cephea	TF	6 months	LVEF <35%
Bapat et al. 2018 (10)	50	14	Single-arm trial	Intrepid	TA	6 months	LVEF < 20%; creatinine > 2.5 mg/dl
Barbanti et al. 2017 (13)	2	1	Observational	HighLife	TA	5 months	NA
Duncan et al. 2017 (14)	5	1	Observational.	Tendyne	TA	2 years	NA
Gössl et al. 2022 (15)	20	8	Single-arm trial and compassionate use	Tendyne	TA	1 year	Absence of severe MAC
Ludwig et al. 2020 (16)	11	1	Observational (compassionate use + post-approval experience)	Tendyne (7) Tiara (4)	TA	6 months	NA
Sorajja et al. 2019–1 (17)	100	24	Single-arm trial	Tendyne	TA	2 years	LVEF < 30%; LVDD > 70, severe pulmonary hypertension
Sorajja et al. 2019–2 (18)	9	5	Observational (compassionate use)	Tendyne	TA	1 year	Absence of severe MAC
Webb et al. 2019 (19)	10	1	Single-arm trial	Sapien M3	TF	30 days	LVEF < 30%; LVDD > 70
Webb et al. 2020 (20)	14	9	Observational (compassionate use + early feasibility)	Evoque	TF	30-days	LVEF < 30%; creatinine > 2.2 mg/dl
Wild et al. 2022 (11)	108	26	Observational	Tendyne	TA	30 days	NA
Zahr et al. 2022 (12)	15	6	Single-arm trial	Intrepid	TF	30 days	LVEF < 25%; LVDD > 75 mm

LVDD, left ventricle diastolic diameter; LVEF, left ventricle ejection fraction; MAC, mitral annular calcification; NA, not available; TA, transapical; TF, transfemoral.

### Baseline features

The main clinical and baseline characteristics for the included studies are shown in Table 2. Twelve studies and 347 patients were included. The weighted mean age was 75.6 years, and 138 (39.8%) were female. The vast majority of patients exhibited at least grade 3+ MR (95.1%), and had mildly reduced ejection fraction (weighted mean left ventricular ejection fraction 47.6%). Most patients had a functional mechanism of MR (58%), and baseline risk for surgical mitral valve replacement was intermediate-high (weighted mean for STS-PROM was 7.1% and for EuroSCORE-2 was 8.5%).

**Table 2:** Clinical characteristics of patients from selected studies.

Study	Number of patients	Age	Female	Atrial fibrillation	Functional MR	≥ Grade 3+ MR	LVEF	STS-PROM	EuroSCORE II
Alperi et al. 2020 (9)	3	79.3 ± 10.3	2 (66.7)	2 (66.7)	0	3 (100)	52.7 ± 6.3	NA	13.8 ± 9
Bapat et al. 2018 (10)	50	72.6 ± 9.4	21 (42)	29 (58)	36 (72)	47 (95.9)	43.4 ± 11.8	6.4 ± 5.5	7.9 ± 6.2
Barbanti et al. 2017 (13)	2	67 ± 8	1 (50)	1 (50)	2 (100)	2 (100)	25	NA	6.7 ± 9
Duncan et al. 2017 (14)	5	73.8 ± 8.7	2 (40)	NA	4 (80)	5 (100)	47.8 ± 10.2	15.4 ± 6.2	16.8 ± 9.9
Gössl et al. 2022 (15)	20	77.6 ± 5.9	11 (55)	13 (65)	NA	20 (100)	58 ± 9	8.1 ± 6.4	NA
Ludwig et al. 2020 (16)	11	78 ± 8.1	8 (72.7)	9 (81.8)	5 (45.5)	11 (100)	44.3 ± 13.3	4.7 ± 3.5	9.8 ± 9
Sorajja et al. 2019–1 (17)	100	75.4 ± 8.1	31 (31)	NA	89 (89)	99 (99)	46.4 ± 9.6	7.8 ± 5.7	NA
Sorajja et al. 2019–2 (18)	9	77 ± 6	4 (44)	NA	0	8 (88)	56 ± 8	7.4 ± 3.6	NA
Webb et al. 2019 (19)	10	76.1 ± 5.5	5 (50)	3 (30)	4 (40)	10 (100)	46.2 ± 11.1	3.8 ± 2.5	5.9 ± 2.2
Webb et al. 2020 (20)	14	83.7 ± 6.7	5 (35.7)	13 (92.9)	3 (21.4)	13 (92.9)	52.5 ± 12.2	4.7 ± 1.3	NA
Wild et al. 2022 (11)	108	75 ± 7	46 (43)	76 (70)	38 (37)	98/104 (94.2)	48 ± 12	7.2 ± 5.3	8.4 ± 6.1
Zahr et al. 2022 (12)	15	79 ± 8.1	2 (13)	6 (40)	NA	14 (100)	49 ± 6.7	5.4 ± 4.1	NA
**Weighted means and CI or proportions**	347	75.6 (75.3–75.8)	138/347 (39.8)	152/233 (65.2)	181/312 (58)	330/347 (95.1)	47.6 (47.2–48)	7.1 (6.9–7.2)	8.5 (8.3–8.7)

CI, confidence interval; LVEF, left ventricle ejection fraction; MR, mitral regurgitation; NA, not available; STS-PROM, society of thoracic surgeons predictive risk of mortality.

Data are expressed as mean ± SD for continuous data, mean (95% confidence interval) for weighted continuous data, and *n* (%) for counts.

### Procedural and 30-day outcomes

Procedural and early outcomes are displayed in Table 3. Technical success was achived for the majority of patients (331/34, 95.4%), and there was a very low rate of conversion to open-heart surgery (1.4%). The rates for 30-day mortality and stroke were 8.4% (29/347) and 2.6% (9/347), respectively. Additionally, the overall rate for life-threatening or major bleeding was 15.6% (29/347) and, for patients with data on acute kidney injury (AKI) (*n* = 310), the rate for this complication was 13.9%.

**Table 3:** Procedural and early outcomes from the studies included for patients undergoing TMVR.

Study	Number of patients	Technical success	Conversion to surgery	Procedural time	30-day mortality	30-day stroke	30-day life-threatening or major bleeding	30-day AKI	30-day LVOT obstruction	30-day ≥ moderate MR	30-day NYHA 3–4
Alperi et al. 2020 (9)	3	3 (100)	0	186 ± 77	0	0	0	1 (33.3)	0	0	0
Bapat et al. 2018 (10)	50	48 (96)	0	NA	7 (14)	2 (4)	9 (18)	5 (10)	0	0/42	10 (23.8)
Barbanti et al. 2017 (13)	2	2 (100)	0	NA	1 (50)	0	0	NA	0	0	0/1
Duncan et al. 2017 (14)	5	4 (80)	0	NA	0	0	0	NA	1 (20)	0	0
Gössl et al. 2022 (15)	20	19 (95)	0	125 ± 39	1 (5)	1 (5)	1 (5)	NA	1 (5)	0	3 (15)
Ludwig et al. 2020 (16)	11	11 (100)	0	NA	0	0	2 (18.2)	1 (9.1)	0	0	3 (27.3)
Sorajja et al. 2019–1 (17)	100	96 (96)	0	136 ± 36	6 (6)	2 (2)	18 (18)	8 (8)	1 (1)	1/97 (1)	19 (19)
Sorajja et al. 2019–2 (18)	9	8 (89)	0	130 ± 44	0	0	1 (11)	2 (22)	1 (11)	0	1 (11)
Webb et al. 2019 (19)	10	9 (90)	0	220 ± 45	0	0	1 (10)	NA	0	1 (10)	1 (10)
Webb et al. 2020 (20)	14	13 (92.9)	1 (7.1)	179.5 ± 38.3	1 (7.1)	2 (14.2)	3 (21.4)	3 (21.4)	1 (7.1)	0/12	2 (18.2)
Wild et al. 2022 (11)	108	104 (96)	3 (3)	130 ± 41	13 (12)	2 (2)	12 (11)	23 (21)	2 (2)	0/105	24/90 (27)
Zahr et al. 2022 (12)	15	14 (93)	1 (7)	166.3 ± 77	0	0	7 (47)	0	2 (13.3)	0/14	2 (14)
**Weighted means and CI or proportions**		331/347 (95.4)	5/347 (1.4)	140 (137–143)	29/347 (8.4)	9/347 (2.6)	54/347 (15.6)	43/310 (13.9)	9/347 (2.6)	2/330 (0.6)	65/328 (19.8)

AKI, acute kidney injury; CI, confidence interval; LVOT, left ventricle outflow tract; MR, mitral regurgitation; NYHA, New York heart association.

Data are expressed as mean ± SD for continuous data, mean (95% confidence interval) for weighted continuous data, and *n* (%) for counts.

**Figure 2:**
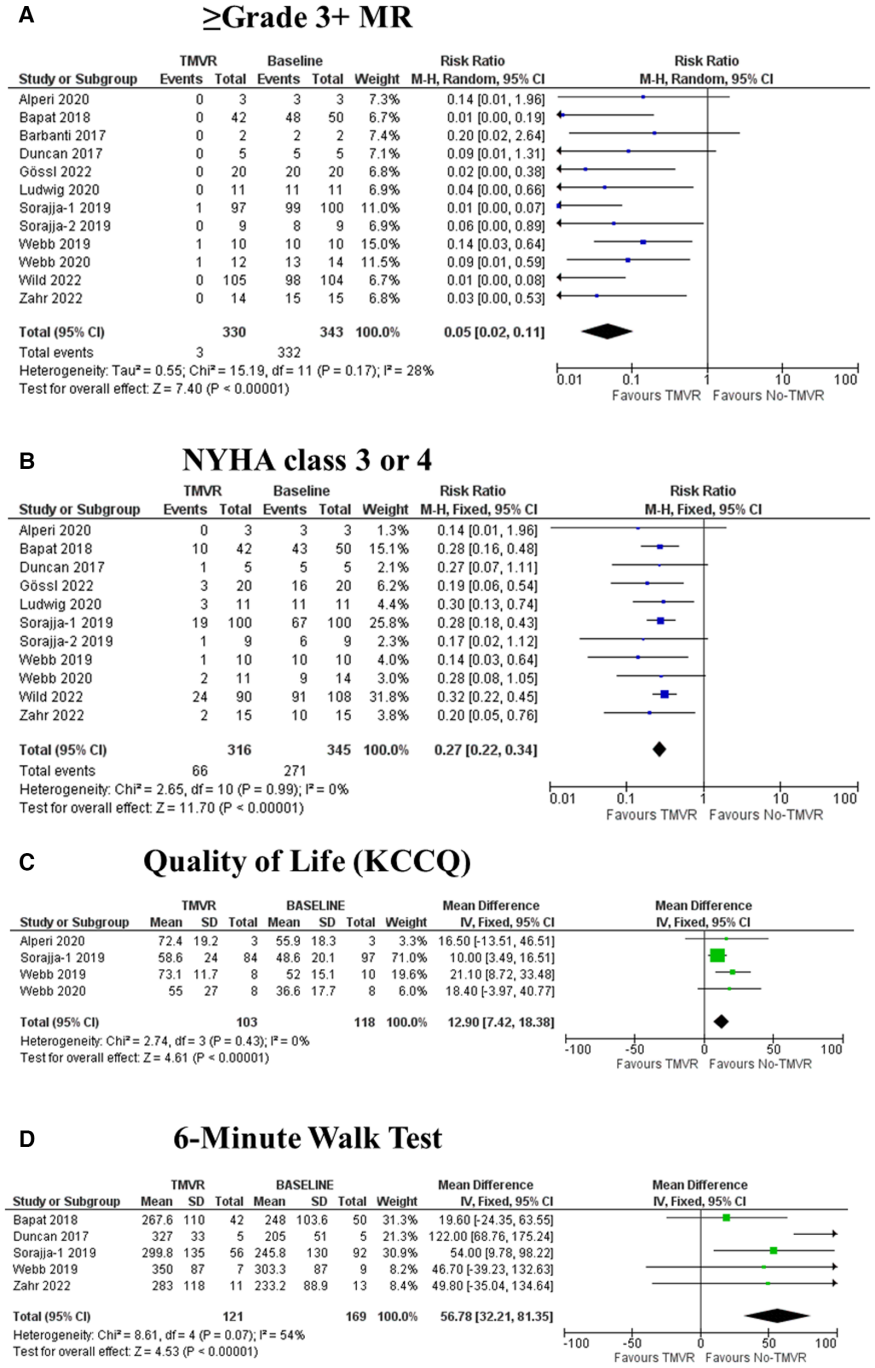
Forest plots reporting the study outcomes evaluated pre- and post-TMVR. IV: inverse variance. KCCQ: Kansas City Cardiomyopathy Questionnaire. M-H: Mantel-Haenszel. MR: mitral regurgitation.

At discharge, a total of 2 out of 333 patients (0.6%) exhibited ≥ grade 3+ MR, compared to 330/347 (95.1%) at baseline. Pooled random-effects results yielded a significant reduction in ≥ grade 3+ MR after the procedure (RR: 0.05; 95% CI: 0.02–0.11; *p* < 0.001) (Figure 2A), and the level of heterogeneity was low (*I*^2 ^= 28%).

### Functional class and quality of life assessment

A total of 316 patients had data on 30-day NYHA class. Of them, a total of 66 (20.9%) exhibited poor functional class (NYHA 3 or 4). Pooled random-effects yielded a significant reduction in 30-day NYHA 3–4 after the intervention (RR: 0.27; 95% CI: 0.22–0.34; *p* < 0.001) (Figure 2B), and there was no heterogeneity (*I*^2 ^= 0%).

A total of 4 studies reported on quality-of-life changes based on the KCCQ. Overall, there was a statistically significant improvement after the intervention compared to baseline, with a pooled fixed-effect mean difference estimate of 12.9 points (95%7.4–18.4, *p* < 0.001) (Figure 2C). No heterogeneity was observed for this endpoint (*I*^2 ^= 0%).

A total of 5 studies reported on exercise capacity based on the 6-minute walk test. Overall, there was a statistically significant increase in the distance walked after the intervention, with a pooled fixed-effects mean difference estimate of 56.8 meters (95% CI 32.2–81.3, *p* < 0.001) (Figure 2D). There was moderate heterogeneity for this endpoint (*I*^2 ^= 54%).

### Mid-term outcomes

Data beyond the 1st month after procedure were available for 8 studies (Table 4). The weighted mean follow-up time was 17.5 months (95% CI 15.8–19.2 months). All-cause mortality and heart failure re-hospitalization were 25.8% (51/198) and 31.3% (51/163), respectively. There was a sustained abolition of MR with none of the patients with long-term echocardiography exhibiting residual MR > mild.

**Table 4:** Long-term outcomes for the studies providing long-term data.

Study	Follow-up	All-cause mortality	HF hospitalization	≥Moderate MR at last echo	NYHA 3–4
Alperi et al. 2020 (9)	6 months	0	0	0	0
Bapat et al. 2018 (10)	6 months	11 (22)	12 (24)	0/42	9/43 (20.9)
Barbanti et al. 2017 (13)	5 months	1	0/1	0/1	0/1
Duncan et al. 2017 (14)	6 years	1 (20)	NA	0/4	0/4
Ludwig et al. 2020 (16)	6 months	2/9 (22)	NA	0/5	1/5 (20)
Gössl et al. 2022 (15)	1 year	8 (40)	6 (30)	0	1/11 (9.1)
Sorajja et al. 2019–1 (17)	1 year	26 (26)	31 (31)	0/44	9/49 (18.4)
Sorajja et al. 2019–2 (18)	1 year	2 (22)	2 (22)	0	1 (11)
**Weighted means and CI or proportions**	17.5 months (15.8–19.2)	51/198 (25.8)	51/163 (31.3)	0/128 (0)	21/125 (16.8)

CI, confidence interval; HF, heart failure; MR, mitral regurgitation; NYHA, New York heart association.

Data are expressed as mean ± SD for continuous data, mean (95% confidence interval) for weighted continuous data, and *n* (%) for counts.

## Discussion

The main findings of this systematic review and meta-analysis can be summarized as follows: (i) among 347 patients comprising the experience with current TMVR devices, there was a high technical success rate (95.4%), and LVOT obstruction and stroke rates were relatively low (2.6% for both); (ii) 30-day mortality was 8.4%, and the rate for early significant bleeding is still of concern (15.6%); (iii) there was a statistically significant reduction in ≥ grade 3+ MR and in the number of patients exhibiting poor functional class (NYHA 3 or 4) after the intervention; and (iii) both quality of live and exercise capacity improved significantly after mitral valve replacement.

There has been a gradual refinement in valve technology since the beginning of the TMVR experience. It consists of ameliorated device deliverability, a reduction in the size of vascular access sheaths, and the adoption of straightforward and more reproducible implantation techniques. This has led to improved clinical outcomes, with a steady reduction in the rates of unsuccessful device implants, early bleeding, stroke, and mortality. As a matter of fact, technical success rates were below 80% for some systems that are no longer under clinical use/evaluation (21), whereas technical success rates were above 95% in our updated systematic review.

Stroke has probably been the most dreadful complication in the transcatheter structural field. Our pooled data show that currently used TMVR devices associate relatively low rates of cerebrovascular complications (2.4%), and these rates range between 0% and 3% for the most recently published studies in the field (12, 15). These promising results highlight the reduction in stroke events when compared to the very early clinical experience in TMVR (22). However, procedural safety still lies way behind in comparison with other percutaneous mitral valve interventions such as TEER, where stroke rates have been reported to be systematically below 1% (23).

Major (and life-threatening) bleeding remains one of the primary shortcomings of TMVR, with pooled rates above 15% in our analysis. This limitation could be partially explained by the learning curve associated with the use of some devices. Notably, many studies had sample sizes below 20 patients; hence, they illustrate the early stages of clinical use for several systems. Besides, the predominant utilization of the transapical route may have played a major role in these findings, as pericardial and thoracotomy-related bleedings are often of concern. Further device and delivery system iterations are needed to increase procedural safety. Besides, wider adoption of TMVR and growing operator experience will help overcome this issue.

In our study, most of the implantations were performed transapically (315/347, 90.78%), which may be mainly explained by the obtention of the CE mark by the Tendyne device system (Abbott, Menlo Park, California). It should be noted that the growing use of the transfemoral approach for some new devices may help overcome the detrimental effects associated with the transapical access, such as pericardial bleeding and myocardial injury. Additionally, the shift from transapical to transfemoral TMVR procedures could be associated with a more straightforward patient recovery translating into a shorter hospital stay. The appearance of new devices purposely designed for transfemoral-transeptal access (e.g., Cephea, Evoque, M3), and the iterations allowing for transfemoral implantations in systems which were firstly developed exclusively for transapical procedures (e.g., Intrepid) are of upmost importance to improve TMVR safety and to facilitate its expansion.

Valve performance and sustained MR abolition are probably the most significant advantages of TMVR over other transcatheter techniques used in MR patients. Accordingly, we have shown a significant MR reduction after the procedure when dichotomizing moderate-severe vs. less than moderate MR, and the number of patients with significant MR despite TMVR is minimal. In addition, these results seem to be maintained over time, as no significant MR was observed at mid-term follow-up echocardiography. The 2-year results of the largest TMVR single-arm trial have been reported, and MR at 2-year follow-up ranged between none-trace (93.2%) and mild (6.8%) (24), with no cases of significant mitral stenosis. Future studies and larger follow-ups are necessary to comprehensively assess valve durability and its performance over time, although these early results are encouraging. It should be noted that these results apply exclusively to patients with severe MR. Some studies have included patients with a certain degree of mitral stenosis, especially those reporting on TMVR in mitral annular calcification (9, 15, 18). However, there are no specific reports for patients with significant mitral stenosis without regurgitation, and the scarce number of patients with concomitant stenosis does not allow for a separate subanalysis. TMVR performance in this setting (isolated mitral stenosis) remains widely unknown.

MR does not exclusively impact life expectancy but also the quality of life and functional capacity. Our study demonstrates a rapid improvement in functional class, quality of life, and exercise capacity early after TMVR, probably based on an effective MR reduction with no need for a sternotomy or major surgery. These findings should be taken into perspective, as patients ultimately treated with TMVR exhibited several comorbidities and advanced age. Therefore, both qualitative and quantitative ameliorations in quality of life are of great clinical relevance.

Despite the findings in our systematic review, some issues remain to be addressed. For instance, screening failure based on anatomical features such as the risk for LVOT obstruction and mitral annular sizing was not systematically reported among the selected studies, but it may have been relatively high. This issue could hamper TMVR applicability in routine clinical practice. Additionally, whether to perform mitral valve repair or replacement when TEER is doable from a technical standpoint is controversial. TMVR outperforms TEER in terms of MR reduction, although it implies a higher stroke and bleeding risk. Besides, long-term complications associated with prosthetic valves, such as infective endocarditis or valve thrombosis (scarcely evaluated in current TMVR studies, ought to the short clinical follow-ups) should be considered. On the other hand, TMVR carries an almost negligible risk for high residual mitral gradients. As previously reported (25), TMVR may be a good alternative when TEER implies a prohibitive increase in diastolic mitral gradient. Ongoing trials comparing TMVR vs. TEER will shed more light on this matter (NCT03433274). For the time being, and given the amount of clinical evidence available for TEER, it seems reasonable to undertake an upfront percutaneous repairment strategy when feasible.

Some limitations merit acknowledgement in our study. First, the nature of the included studies was observational for the vast majority, and no control group was used for the trials included. Therefore, TMVR performance vs. medical therapy or other invasive techniques should be further evaluated. Publication bias is a potential limitation as for any meta-analysis. However, Eggeŕs regression analysis and funnel-plots did not suggest its presence (Supplementary Material Table S1 and Supplementary Material Figures 1–3). Furthermore, the lack of substantial clinical experience with some devices might have significantly impacted pooled outcomes, as well as the predominant use of the Tendyne device among the patients included. Future systematic analysis as TMVR experience grows over the following years will be paramount to monitor the field's evolution.

In conclusion, TMVR with currently available systems yields a significant MR reduction and an early improvement in functional class and quality of life, but with the trade-off of relatively high major bleeding rates.

## Data Availability

The raw data supporting the conclusions of this article will be made available by the authors, without undue reservation.
