# Supplementary material for: Current status of transcatheter mitral valve replacement: systematic review and meta-analysis
Source: Front Cardiovasc Med. 2023 May 10;10:1130212. doi: 10.3389/fcvm.2023.1130212 (PMC10206247; doi:10.3389/fcvm.2023.1130212)

**Supplemental Table 1**: Egger regression p values for the pooled comparisons.

| **Comparisons** | **Egger regression p value** |
| --- | --- |
| ≥grade 3+ MR | 0.14 |
| NYHA class 3-4 | 0.32 |
| KCCQ | 0.25 |
| 6-minute walk test | 0.64 |

KCCQ: Kansas City Cardiomyopathy Questionnaire. MR: Mitral regurgitation.

**Supplemental Figure 1**: Funnel plot for the endpoint ≥grade 3+ MR after TMVR.


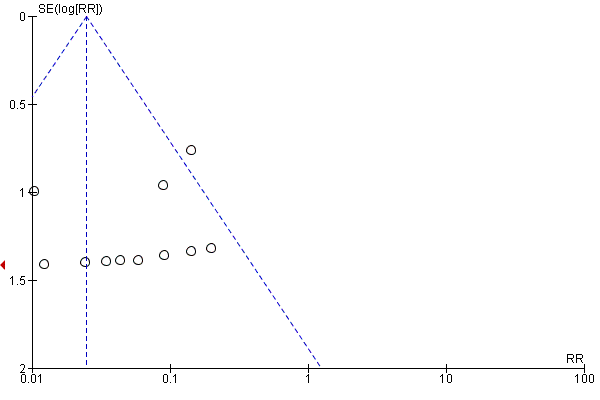


**Supplemental Figure 2**: Funnel plot for the endpoint NYHA class 3 or 4 after TMVR.


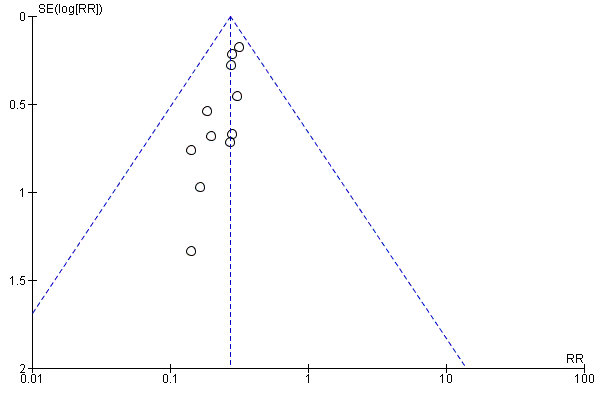


**Supplemental Figure 3**: Funnel plot for the endpoint KCCQ score after TMVR.


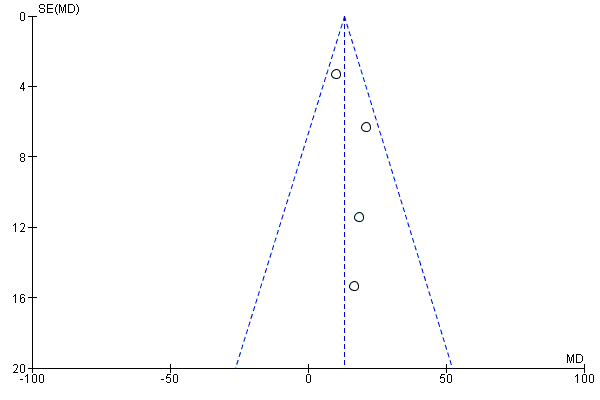

Supplement: Supplementary file 1 [file Datasheet1.docx]
